# Uncovering the Molecular Drivers of NHEJ DNA Repair-Implicated Missense Variants and Their Functional Consequences

**DOI:** 10.3390/genes14101890

**Published:** 2023-09-29

**Authors:** Raghad Al-Jarf, Malancha Karmakar, Yoochan Myung, David B. Ascher

**Affiliations:** 1 Structural Biology and Bioinformatics, Department of Biochemistry, University of Melbourne, Parkville, VIC 3052, Australiamkarmakar@student.unimelb.edu.au (M.K.);; 2 Systems and Computational Biology, Bio21 Institute, University of Melbourne, Parkville, VIC 3052, Australia; 3 Computational Biology and Clinical Informatics, Baker Heart and Diabetes Institute, Melbourne, VIC 3004, Australia; 4 School of Chemistry and Molecular Biosciences, University of Queensland, St. Lucia, QLD 4072, Australia

**Keywords:** non-homologous end joining, DNA repair genes, DNA Ligase IV, DNA-PKcs, KU70/80, XRCC4, variant analysis, protein-protein interactions

## Abstract

Variants in non-homologous end joining (NHEJ) DNA repair genes are associated with various human syndromes, including microcephaly, growth delay, Fanconi anemia, and different hereditary cancers. However, very little has been done previously to systematically record the underlying molecular consequences of NHEJ variants and their link to phenotypic outcomes. In this study, a list of over 2983 missense variants of the principal components of the NHEJ system, including DNA Ligase IV, DNA-PKcs, Ku70/80 and XRCC4, reported in the clinical literature, was initially collected. The molecular consequences of variants were evaluated using in silico biophysical tools to quantitatively assess their impact on protein folding, dynamics, stability, and interactions. Cancer-causing and population variants within these NHEJ factors were statistically analyzed to identify molecular drivers. A comprehensive catalog of NHEJ variants from genes known to be mutated in cancer was curated, providing a resource for better understanding their role and molecular mechanisms in diseases. The variant analysis highlighted different molecular drivers among the distinct proteins, where cancer-driving variants in anchor proteins, such as Ku70/80, were more likely to affect key protein–protein interactions, whilst those in the enzymatic components, such as DNA-PKcs, were likely to be found in intolerant regions undergoing purifying selection. We believe that the information acquired in our database will be a powerful resource to better understand the role of non-homologous end-joining DNA repair in genetic disorders, and will serve as a source to inspire other investigations to understand the disease further, vital for the development of improved therapeutic strategies.

## 1. Introduction

Maintaining the integrity of the genome is crucial for any organism’s survival [1]. Double-strand breaks (DSBs) are deemed as one of the most harmful forms of DNA damage since, if left unrepaired, they can result in cell death, or chromosomal rearrangements if inappropriately repaired, leading to cancer [2]. Nonhomologous DNA end joining (NHEJ) is one of the main DSB repair pathways used to repair DNA DSBs in mammalian cells and occurs throughout the cell cycle [3].

The primary participating factors in NHEJ DNA repair machinery include Ku70/Ku80 heterodimers, DNA-PKcs, XLF, XRCC4, and DNA Ligase IV. The Ku70/Ku80 heterodimer binding to the broken DNA initiates the NHEJ repair machinery. Hence, this recruits DNA-PK, whose autophosphorylation is vital for NHEJ. After DSB end-processing, Ligase IV interacts with XRCC4 and XLF to form an NHEJ-specific Ligase [4]. 

Variants have been defined in multiple components of the NHEJ DNA repair pathway, including *PRKDC* (encoding DNA-PKcs), *XRCC4*, *XRCC5* (encoding Ku80)*, XRCC6* (encoding Ku70) and *LIG4* (encoding DNA Ligase IV) [5]. These variants have been associated with various human syndromes, including microcephaly [6], severe combined immunodeficiency (SCID) [7], growth delay, Fanconi anemia [8], and different hereditary cancers [9,10]. Additionally, it has been demonstrated by a significant amount of genetic evidence that the loss or variation of the core NHEJ players leads to increased genomic instability and the development of cancer [11]

Numerous studies have sought to identify genetic single nucleotide polymorphisms associated with carcinogenesis in the core NHEJ factors [12,13,14,15]. Notably, many of these studies had small patient sample sizes and needed to be subsequently verified. Indeed, many of the NHEJ proteins are well described; however, information about the molecular consequences of missense variants in NHEJ’s main components has yet to be fully characterized by a single source. Thus, this information is scattered throughout the literature.

Previously, we have demonstrated that computational approaches can be applied for a more profound understanding of the effects of missense variants on the 3D structure of the protein to elucidate the molecular mechanisms underlying the disease and improve the prediction of the disease prognosis [16,17,18].

We prioritized four NHEJ core factors, Ku70/80, Ligase IV, DNA-PKcs, and XRCC4, for our computational analysis due to the relatively high concentration of cancer-causing missense variants distributed in these factors. To that end, we characterized and analyzed cancer-causing missense variants’ structural and functional consequences and compared them statistically to those caused by nonpathogenic (population) variants. In addition to providing the most exhaustive list of missense variants for NHEJ core components, this study incorporates a methodology for exploring and analyzing these variants to better understand vital mechanisms of genetic disorders.

## 2. Methods

### 2.1. Data Collection

As a starting point, the disease-causing (clinical) missense variants were collected from the COSMIC [19] database that incorporates somatic variants in human cancer. These variants were first curated in 2019 and updated in 2022. 

The core NHEJ factors XRCC4 (n = 67) Ligase IV (n = 259), Ku70/80 heterodimer (n = 346) and DNA-PKcs (n = 654) were favored owing to their strong associations with disease and their respective enrichments in missense variants. Additionally, we collected a set of nonpathogenic variants based on population variation acquired using gnomAD [20] V.2.1.1; these variants were annotated using the Ensembl Variant Effect Predictor (VEP) [21] V.95. At this stage, we removed those variants that showed inconsistent variantal consequences across both the COSMIC and gnomAD databases to reduce the potential for misunderstanding, and any remaining population variants in XRCC4 (n = 83) DNA Ligase IV(n = 444), Ku70/80 heterodimer (n = 380) and DNA-PKcs (n = 1483) were regarded as nonpathogenic.

### 2.2. NHEJ Structural Curation

It was possible to obtain the experimental crystal structure of the Ku 70/80 heterodimer (PDB ID: 6ERG [22]) bound to DNA and XLF generated at a resolution of 2.90 Å. Structural pre-processing and minimization was performed using Maestro V.11.4 to fill in missing atoms and residues and to remove atomic clashes. This structure was used to calculate Ku70/80 variant features, while the AlphaFold2 structure of Ku70/80 was used to obtain the predicted local distance difference test (pLDDT) scores. DNA-PKcs’ crystal structure (PDB ID: 5Y3R [23]) was available bound to Ku70/80, and it was used for calculating the variant features for DNA-PKcs. Similar to Ku70/80, the structure obtained using AlphaFold2 of DNA-PKcs was used to calculate pLDDT scores. As RCSB PDB lacks full experimental structures for LIG4 and XRCC4, AlphaFold2 [24] was used to generate full structures for these proteins. One experimental structure of LIG4 bound to DNA was found (PDB: 6BKG, residues 1–620) and used for calculating the variantal features, specifically (changes in nucleic acid (DNA) affinity).

### 2.3. Feature Engineering

Biophysical properties of proteins were calculated by considering changes in protein–protein interaction, function and conservation, stability and dynamics, and local residue environment. Wildtype residue environment parameters were investigated, including relative solvent accessibility (RSA), backbone phi and psi angles, and residue depth. Several conservation-based features were incorporated, including rates of residue evolution, ConSurf [25], and deleteriousness predictions using Polyphen-2 [26], SNAP2 [27], PROVEAN [28], MTR3D [29], Envision [30], and SIFT [31]. Further, we considered evolutionary substitution matrices such as PAMs and BLOSUMs. Alphafold2 pLDDT scores [24] were also assessed for determining residue localizations within disordered regions.

We conducted *in silico* biophysical measurements based on mCSM-Stability [32], DynaMut2 [33], DynaMut [34], SDM [35], and DUET [36] to predict variants’ changes in stability and dynamics. Also, we calculated variantal effects on protein–protein interactions via mCSM-PPI [37] and mCSM-PPI2 [38], along with distances to the interface. The calculations of protein interactions included gene-dependent Ku heterodimer, DNA-PKcs and LIG4 bound to DNA. The associated impacts of these bindings on affinity were calculated for the experimental structures 6BKG and 5Y3R with mCSM-NA [39]. For DNA-PKcs, the distance to ATP was measured. Using Arpeggio [40], we assessed the effects of variant on local molecular interactions.

### 2.4. Qualitative and Statistical Analysis

We compared the consequences of pathogenic and nonpathogenic variants on the calculated features using Welch’s two-tailed t-test to determine potential molecular drivers in the NHEJ repair machinery. To evaluate features as potential molecular drivers, we looked for statistically significant differences between the two classes (*p*-value < 0.05).

A comparison of individual variants in terms of heterodimer affinity (mCSM-PPI and mCSM-PPI2), protein stability (DynaMut2), and vibrational entropy (ENCoM value, obtained using DynaMut) was performed, as previously described, based on their 0structural localization. Variants of KU70/80 were assessed based on heterodimer affinity changes rather than stability changes since even minor changes at the heterodimer interface can significantly contribute to pathogenicity. It is noteworthy that only variants located within a 10 Å of the protein–protein interface of the Ku70/80 heterodimer were examined, since heterodimer affinity has been regarded to subside over distance. 

All measures represented as a difference in Gibbs free energy of folding (ΔΔG, in kcal/mol) were assessed based on their magnitude and direction, low (0.05 ≤ |ΔΔG| < 0.5), intermediate (0.5 ≤ |ΔΔG| < 1) or high (|ΔΔG| ≥ 1), further highlighting each variant’s main molecular consequence.

### 2.5. Model Training

Our final analysis used the ensemble algorithm ExtraTrees (with 100 trees) within Sci-kit Learn V.0.20.3. [41] to test the predictability of important features for phenotyping variants. A comparison of the performance of all features and subsets of important features in phenotyping was conducted, with important features highlighted from each model.

## 3. Results

### 3.1. Data Curation and Variant Distribution of NHEJ Principal Components

The final curated database was acquired from COSMIC and gnomAD and incorporates a total of 1326 pathogenic and 2390 nonpathogenic missense variants in NHEJ main factors, spread across five genes, summarised in online Supplemental Table S1. Although missense variants in the main components of NHEJ repair machinery are not the only cause of the disease (cancer), computational approaches, such as those accounting for protein structural consequences, can effectively analyse these types of genetic variation.

Accordingly, we identified potential molecular drivers of disease by applying our computational analysis pipeline to the most mutated genes in NHEJ DNA repair machinery: LIG4, Ku70/80 (*XRCC5/6*), DNA-PKcs (*PRKDC*), and *XRCC4*. An overview of the phenotypes collected for each of the NHEJ core components is described in Table 1. 

Next, we visualized the distributions of the missense variants within the structures of NHEJ principal components (Supplemental Figure S1), which illustrated that cancer-causing (pathogenic) variants were widely distributed across each gene of the NHEJ core components and their subsequent 3D structures without a specific localization. Similar patterns were observed for the population variants (nonpathogenic) within each of the principal NHEJ players.

### 3.2. Identifying Molecular Drivers in Ku70/80 Heterodimer

**Figure 1:**
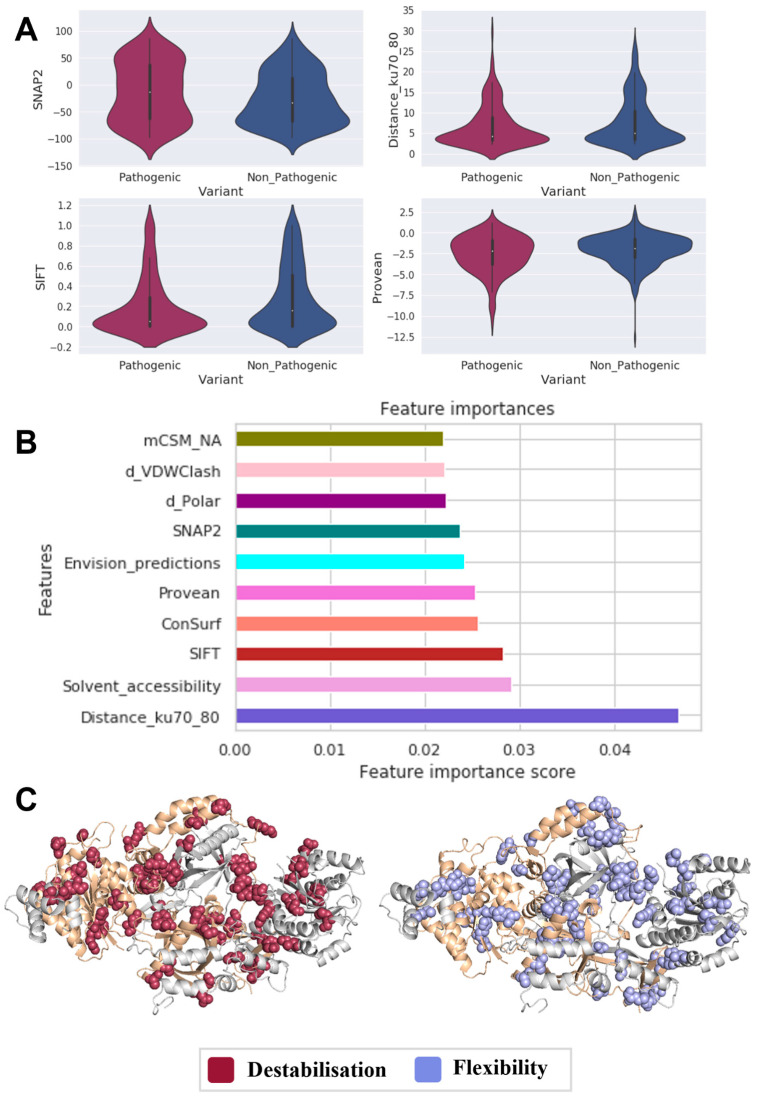
Main drivers of Ku70/80 pathogenicity. Based on statistically significant features (**A**), interaction profiles associated with variants play an essential role in Ku-mediated tumorigenesis. Supervised machine learning (**B**) confirmed the high predictive potential of the changes in the protein–protein interface of the Ku heterodimer. Through mapping variants on the heterodimer structure (**C**), we were able to verify that stability plays a critical role in disease (red), as do conformational changes (light blue).

A comparison of the molecular effects of Ku70/80 pathogenic (n = 346) with nonpathogenic (n *=* 380) variants (Figure 1A) revealed that pathogenic variants were more likely to be found close to the protein–protein interface, leading to a disruption of the interaction between the KU70/80 heterodimer (Distance_Ku70_80 *p*-value: 0.043). In addition, as estimated by measures of functional deleteriousness (SNAP2 *p*-value: 0.022, PROVEAN *p*-value: 0.017, SIFT *p*-value: 0.003), pathogenic variants tend to occur at functionally essential protein regions.

Based on these effects, we developed a predictor that could correctly identify 91% of pathogenic variants and 96% of nonpathogenic variants. As a result of our predictor predictions, Distance_Ku70_80 has been deemed the most significant pathogenicity driver (contributed the most by 4%, Figure 1B). According to these observations, tumorigenesis is primarily associated with a Ku70/80 function disruption, where pathogenic variants are localized within the protein–protein interface.

As a final analysis, to determine the main drivers of pathogenicity in Ku70/80, we analyzed each pathogenic variant structurally (Figure 1C). We found that 54% of these variants decrease stability, and 60% reduce protein–protein interactions within the Ku heterodimer. The findings indicate that, in addition to reducing stability, Ku-mediated tumorigenesis is caused by a decrease in protein–protein interactions and conformational changes within the heterodimer.

### 3.3. Identifying Molecular Drivers of Pathogenicity in DNA-PKcs

An analysis of DNA-PKcs pathogenic variants (n = 654) compared to nonpathogenic ones (n = 1483) showed that pathogenic variants were more likely to be found in functionally important and intolerant regions undergoing purifying selection based on conservation (PAM30 *p*-value: 3 × 10^−5^) and function (MTR score *p*-value: 0.010) calculations for proteins (Supplemental Figure S2A).

As a result of this localization, pathogenic variants were likewise highly likely to be solvent-accessible (RSA *p*-value: 0.004) and, hence, reactive towards binding partners. Regarding ligand binding (ATP), pathogenic variants were particularly clustered near the ATP binding sites (*p*-value: 0.004). Closeness to ATP binding implies that ATP-mediated changes in catalytic DNA-PKcs activity likely drive pathogenicity.

A machine learning-based predictor was trained using all significant features, which correctly classified 97% of pathogenic variants and 94% of nonpathogenic variants. Based on the various contributors to these predictions (Supplemental Figure S2B), it was found that changes in ATP-binding affinity (distance to ATP) contributed the most (16%). In contrast, the MTR score contributed substantially (8%). We also investigated changes in the DNA affinity of the DNA-bound structure (PDB 5Y3R, residues 1503–1538) caused by a subset of three pathogenic and 10 nonpathogenic variants (Supplemental Table S2). No notable differences between phenotypic classes were observed besides the significant enrichment of nonpathogenic variants. It is suggested, however, that DNA-mediated effects are not important drivers of tumorigenesis as the DNA-binding region within DNA-PKcs is enriched in nonpathogenic variants.

### 3.4. Uncovering Molecular Drivers in LIG4

**Figure 2:**
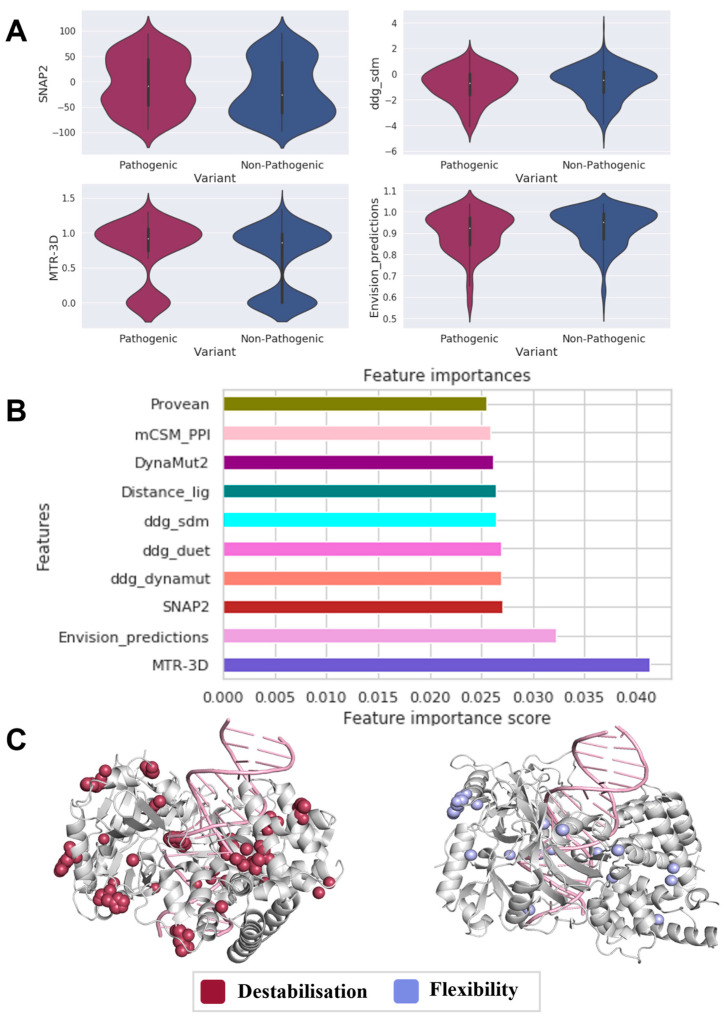
Main drivers of LIG4 pathogenicity. The statistically significant features (**A**) determined by Welch’s sample t-test revealed that LIG4-mediated carcinogenesis is driven by both stability and MTR3D, which signifies functional deleteriousness, as verified via supervised machine learning (**B**), which had the most considerable predictive power. Most pathogenic variants (**C**) result in destabilizing (red) or increasing flexibility (light blue), providing further evidence of conformational effects.

When analyzing the molecular consequences of pathogenic (n = 259) variants in comparison to nonpathogenic (n = 444) variants in DNA LIG4 (Figure 2A), we observed that pathogenic variants tend to cluster in functionally essential regions of the protein (MTR-3D *p*-value: 7 × 10^−5^, SNAP2 *p*-value: 0.023, Envision score *p*-value: 0.009). Additionally, it was observed that pathogenic variants reduce protein stability (ΔΔG-sdm *p*-value: 0.044).

The phenotypes of all pathogenic and nonpathogenic variants in our dataset could be predicted using a machine-learning analysis combining these influential molecular descriptors. For the predictions, the developed classifier used functional scores represented by MTR-3D (4%), Envision (3.2%), SNAP2 (2.7%), and ΔΔG-sdm (2.6%, Figure 2B), further highlighting their involvement in pathogenicity.

Using changes in stability and vibrational entropy to analyze pathogenic variant in LIG4 (Figure 2C), we found that most variants were associated with increased flexibility (23%) or protein destabilization (30%), further establishing the role of protein conformational changes in tumorigenesis and pathogenicity of LIG4.

### 3.5. Uncovering Molecular Drivers in XRCC4

**Figure 3:**
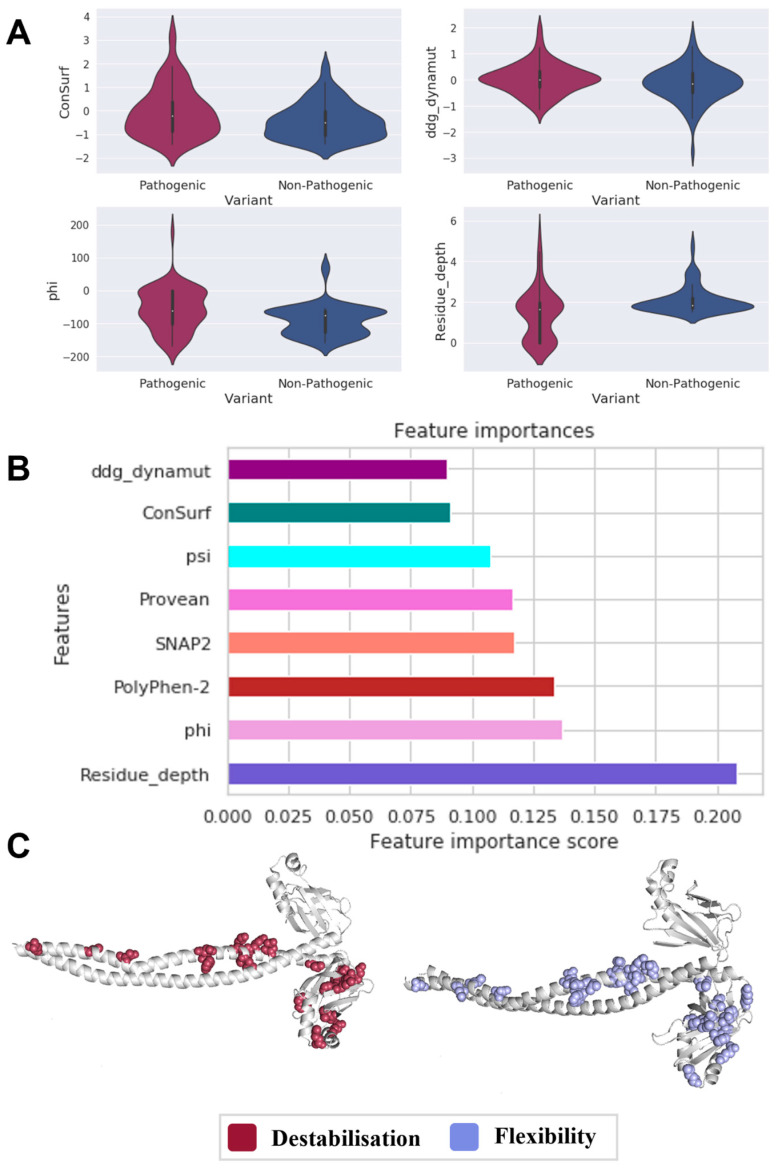
Main drivers of XRCC4 pathogenicity. Welch’s sample t-tests identified statistically significant features (**A**) that suggested protein conformation plays an essential role in XRCC4-mediated tumorigenesis, which may expose key residues close to the protein surface, as indicated by supervised machine learning (**B**). Residue depth had the most considerable predictive power. As we mapped variants in 3D (**C**), we were also able to establish the role of stability and conformational changes in disease (displayed in red and light blue)**.**

Although comparable drivers of pathogenicity were observed in XRCC4 (Figure 3A), as expressed by protein conservation (ConSurf *p*-value: 0.016), distinctive mechanisms for variant localization were seen. The distribution of phi angles (phi *p*-value: 0.007) for pathogenic variants (n = 67) was more distinct than that for nonpathogenic ones (n = 83); they tend to cluster at the core of the protein (residue depth *p*-value: 1.6 × 10^−5^). In addition, variantal changes in stability highlighted that pathogenic variants in XRCC4 are highly destabilizing (ΔΔG_dynamut *p*-value: 0.042). 

When we trained a machine learning-based predictor by combining all of these significant features, 95% of pathogenic and 81% of nonpathogenic variants were correctly classified. The local residue environment represented by residue depth contributed the most towards the predictor predictions, followed by phi angle (17.5%)**,** ConSurf (9%) and ddg_dynamut (9%) (Figure 3B). It is evident from the highlighted results that variant localization explains how different protein conformational states result in functional changes that are essential for elucidating disease.

When pathogenic variant consequences on the protein structure were examined in terms of protein stability and vibrational entropy (Figure 3C), it was found that, in XRCC4, pathogenic variants mainly cause clinical phenotypes by destabilizing the protein (35.8%), as well as causing a rise in protein flexibility (43%), indicating that protein conformational changes contribute to disease development as precursor mechanisms to carcinogenesis.

## 4. Discussion

Our work comprehensively analyzes the consequences of missense variants driving carcinogenesis in the core players of the NHEJ DNA repair machinery, Ku70/80, XRCC4, Ligase IV and DNA-PKcs. Despite being involved in distinctive physiological functions, each of these genes shows standard molecular drivers of disease. Mainly, according to our predictors, all pathogenic variants were functionally deleterious across three core components of NHEJ, Ku70/80, DNA-PKcs and LIG4, while those in XRCC4 localized in areas with a low evolution rate (ConSurf). However, when viewing pathogenic and nonpathogenic variants within protein structures, all variants were widely distributed across the whole structure, with no particular domain localization. 

Other distinctions between the five genes were associated with their unique biological functions. Specifically, pathogenic variants of LIG4 and XRCC4 reduced protein stability, consistent with our previous findings [5].

In addition, Ku70/80 interaction measurements suggest that protein–protein affinity change is crucial for Ku70/80 function, as pathogenic variants cluster near the protein interface and reduce the heterodimer binding. On a larger variant dataset, this study demonstrated the importance of protein–protein interaction affinity in Ku70/80-mediated carcinogenesis, which has been briefly investigated and implicated in destabilizing Ku’s carboxy-terminal arm region that plays an essential role in heterodimerization [42].

Interestingly, when supervised machine learning was used to fit the data, these affinity changes had the highest phenotypic prediction potential. This observation suggests that these changes may play a role in tumorigenesis and pathogenesis. 

Nevertheless, we could not observe conclusive impacts on nucleic acid affinity caused by pathogenic variants when examining the interaction profile of Ku70/80, DNA-PKcs and DNA Ligase IV variants to nucleic acids due to a need for more sufficient data. Despite extensive data curation, pathogenic variants were not detected within nucleic acid binding regions, suggesting that these interactions are crucial for transcription.

Regarding the four protein structures, the molecular drivers identified can be interpreted as a cause of cancer progression and genomic instability. Our work emphasizes how protein–protein affinity change plays a crucial role in Ku70/80-mediated disease, in which protein–protein affinity change presents the best predictability of classifying variants using machine learning. Among the robust predictive features of DNA-PKcs was the distance to ATP and MTR, since pathogenic variants tend to be found closer to ATP binding and intolerant regions undergoing purifying selection. Protein function consequences best indicated the LIG4 variant phenotype. Lastly, when viewing XRCC4, variant phenotype was best predicted by the residue depth, as pathogenic variants were found to occur at the protein core. A closer look at molecular changes revealed several disease mechanisms associated with carcinogenesis.

Over 2983 missense variants are listed in our final database (online Supplemental Table S1), making it the most comprehensive list of NHEJ missense variants available. Cancer patients have also been diagnosed with non-missense variants, such as indels; however, in our work, we focused on missense variants, since structure-based techniques can be used to analyze them with a high throughput, so the three-dimensional consequences of a variant can be adequately considered.

Our database represents the present landscape of missense variants in the NHEJ repair machinery. As missense variants are readily detected in the clinic, cross-referencing variants with our resource can help in the early detection of cancer risk, allowing for the development of therapeutic strategies to slow the disease’s progression. In silico simulations of variantal change can be used to gain insight into disease mechanisms across various genes, as demonstrated in this work using LIG4, Ku70/80 (*XRCC5/6*), DNA-PKcs (*PRKDC*), and *XRCC4*. We have gained insight into disease development across the four proteins by combining structure-based estimators. Furthermore, since cancer is a complex disease with multiple aspects, the structural insights regarded in this study and the implications that may follow may be used to identify effective anticancer treatments.

## Figures and Tables

**Table 1:** Data curation of NHEJ variants.

Protein	Class	n
Ku70/80 heterodimer	Pathogenic	346
	Nonpathogenic	380
DNA-PKcs	Pathogenic	654
	Nonpathogenic	1483
DNA Ligase IV	Pathogenic	259
	Nonpathogenic	444
XRCC4	Pathogenic	67
	Nonpathogenic	83

## Data Availability

This article includes all data relevant to the study, or the data are provided as supplementary information. A list of all the data collected and generated in this study can be found at http://biosig.unimelb.edu.au/strunhej (accessed on 28 September 2023).

## Supplementary Materials

The following supporting information can be downloaded at: https://www.mdpi.com/article/10.3390/genes14101890/s1, Figure S1: An overview of mutation distributions in NHEJ core components; Figure S2: Main drivers of DNA-PKcs pathogenicity; Table S1: NHEJ mutation curated database; Table S2: Mutation effects on DNA affinity in DNA-PKcs;.

## References

[1] Yin M., Hong F., Wang Q.-E. (2022). DNA Damage Response and Cancer Metastasis: Clinical Implications and Therapeutic Opportunities. Metastasis.

[2] Trenner A., Sartori A.A. (2019). Harnessing DNA double-strand break repair for cancer treatment. Front. Oncol..

[3] Chang H.H., Pannunzio N.R., Adachi N., Lieber M.R. (2017). Non-homologous DNA end joining and alternative pathways to double-strand break repair. Nat. Rev. Mol. Cell Biol..

[4] Yano K.-I., Morotomi-Yano K., Adachi N., Akiyama H. (2009). Molecular mechanism of protein assembly on DNA double-strand breaks in the non-homologous end-joining pathway. J. Radiat. Res..

[5] Murray J.E., Van Der Burg M., IJspeert H., Carroll P., Wu Q., Ochi T., Leitch A., Miller E.S., Kysela B., Jawad A. (2015). Mutations in the NHEJ component XRCC4 cause primordial dwarfism. Am. J. Hum. Genet..

[6] Rosin N., Elcioglu N.H., Beleggia F., Isgüven P., Altmüller J., Thiele H., Steindl K., Joset P., Rauch A., Nürnberg P. (2015). Mutations in XRCC4 cause primary microcephaly, short stature and increased genomic instability. Hum. Mol. Genet..

[7] Gao Y., Chaudhuri J., Zhu C., Davidson L., Weaver D.T., Alt F.W. (1998). A targeted DNA-PKcs-null mutation reveals DNA-PK-independent functions for KU in V (D) J recombination. Immunity.

[8] Nie Y., Li Y., Li X., Wilson A.F., Pang Q. (2019). The non-homologous end-joining activity is required for *Fanconi anemia* fetal HSC maintenance. Stem Cell Res. Ther..

[9] Woodbine L., Gennery A.R., Jeggo P.A. (2014). The clinical impact of deficiency in DNA non-homologous end-joining. DNA Repair.

[10] Bau D.-T., Fu Y.-P., Chen S.-T., Cheng T.-C., Yu J.-C., Wu P.-E., Shen C.-Y. (2004). Breast cancer risk and the DNA double-strand break end-joining capacity of nonhomologous end-joining genes are affected by BRCA1. Cancer Res..

[11] Caracciolo D., Riillo C., Di Martino M.T., Tagliaferri P., Tassone P. (2021). Alternative Non-Homologous End-Joining: Error-Prone DNA Repair as Cancer’s Achilles’ Heel. Cancers.

[12] Sishc B.J., Davis A.J. (2017). The role of the core non-homologous end joining factors in carcinogenesis and cancer. Cancers.

[13] Ferguson D.O., Sekiguchi J.M., Chang S., Frank K.M., Gao Y., DePinho R.A., Alt F.W. (2000). The nonhomologous end-joining pathway of DNA repair is required for genomic stability and the suppression of translocations. Proc. Natl. Acad. Sci. USA.

[14] Gu Y., Jin S., Gao Y., Weaver D.T., Alt F.W. (1997). Ku70-deficient embryonic stem cells have increased ionizing radiosensitivity, defective DNA end-binding activity, and inability to support V (D) J recombination. Proc. Natl. Acad. Sci. USA.

[15] Nussenzweig A., Sokol K., Burgman P., Li L., Li G.C. (1997). Hypersensitivity of Ku80-deficient cell lines and mice to DNA damage: The effects of ionizing radiation on growth, survival, and development. Proc. Natl. Acad. Sci. USA.

[16] Portelli S., Phelan J.E., Ascher D.B., Clark T.G., Furnham N. (2018). Understanding molecular consequences of putative drug resistant mutations in Mycobacterium tuberculosis. Sci. Rep..

[17] Portelli S., Barr L., de Sá A.G., Pires D.E., Ascher D.B. (2021). Distinguishing between PTEN clinical phenotypes through mutation analysis. Comput. Struct. Biotechnol. J..

[18] Airey E., Portelli S., Xavier J.S., Myung Y.C., Silk M., Karmakar M., Velloso J.P., Rodrigues C.H., Parate H.H., Garg A. (2021). Artificial Neural Networks.

[19] Tate J.G., Bamford S., Jubb H.C., Sondka Z., Beare D.M., Bindal N., Boutselakis H., Cole C.G., Creatore C., Dawson E. (2019). COSMIC: The catalogue of somatic mutations in cancer. Nucleic Acids Res..

[20] Karczewski K.J., Francioli L.C., Tiao G., Cummings B.B., Alföldi J., Wang Q., Collins R.L., Laricchia K.M., Ganna A., Birnbaum D.P. (2020). The mutational constraint spectrum quantified from variation in 141,456 humans. Nature.

[21] McLaren W., Gil L., Hunt S.E., Riat H.S., Ritchie G.R., Thormann A., Flicek P., Cunningham F. (2016). The ensembl variant effect predictor. Genome Biol..

[22] Nemoz C., Ropars V., Frit P., Gontier A., Drevet P., Yu J., Guerois R., Pitois A., Comte A., Delteil C. (2018). XLF and APLF bind Ku80 at two remote sites to ensure DNA repair by non-homologous end joining. Nat. Struct. Mol. Biol..

[23] Yin X., Liu M., Tian Y., Wang J., Xu Y. (2017). Cryo-EM structure of human DNA-PK holoenzyme. Cell Res..

[24] Jumper J., Evans R., Pritzel A., Green T., Figurnov M., Ronneberger O., Tunyasuvunakool K., Bates R., Žídek A., Potapenko A. (2021). Highly accurate protein structure prediction with AlphaFold. Nature.

[25] Ashkenazy H., Abadi S., Martz E., Chay O., Mayrose I., Pupko T., Ben-Tal N. (2016). ConSurf 2016: An improved methodology to estimate and visualize evolutionary conservation in macromolecules. Nucleic Acids Res..

[26] Adzhubei I., Jordan D., Sunyaev S. (2013). Predicting functional effect of human missense mutations using PolyPhen-2. Current Protocols in Human Genetics.

[27] Hecht M., Bromberg Y., Rost B. (2015). Better prediction of functional effects for sequence variants. BMC Genom..

[28] Choi Y., Chan A.P. (2015). PROVEAN web server: A tool to predict the functional effect of amino acid substitutions and indels. Bioinformatics.

[29] Silk M., Pires D.E., Rodrigues C.H., D’Souza E.N., Olshansky M., Thorne N., Ascher D.B. (2021). MTR3D: Identifying regions within protein tertiary structures under purifying selection. Nucleic Acids Res..

[30] Gray V.E., Hause R.J., Luebeck J., Shendure J., Fowler D.M. (2018). Quantitative Missense Variant Effect Prediction Using Large-Scale Mutagenesis Data. Cell Syst..

[31] Ng P.C., Henikoff S. (2003). SIFT: Predicting amino acid changes that affect protein function. Nucleic Acids Res..

[32] Pires D.E., Ascher D.B., Blundell T.L. (2014). mCSM: Predicting the effects of mutations in proteins using graph-based signatures. Bioinformatics.

[33] Rodrigues C.H.M., Pires D.E.V., Ascher D.B. (2021). DynaMut2: Assessing changes in stability and flexibility upon single and multiple point missense mutations. Protein Sci..

[34] Rodrigues C.H., Pires D.E., Ascher D.B. (2018). DynaMut: Predicting the impact of mutations on protein conformation, flexibility and stability. Nucleic Acids Res..

[35] Pandurangan A.P., Ochoa-Montaño B., Ascher D.B., Blundell T.L. (2017). SDM: A server for predicting effects of mutations on protein stability. Nucleic Acids Res..

[36] Pires D.E., Ascher D.B., Blundell T.L. (2014). DUET: A server for predicting effects of mutations on protein stability using an integrated computational approach. Nucleic Acids Res..

[37] Rodrigues C.H., Pires D.E., Ascher D.B. (2021). mmCSM-PPI: Predicting the effects of multiple point mutations on protein–protein interactions. Nucleic Acids Res..

[38] Rodrigues C.H., Myung Y., Pires D.E., Ascher D.B. (2019). mCSM-PPI2: Predicting the effects of mutations on protein–protein interactions. Nucleic Acids Res..

[39] Pires D.E.V., Ascher D.B. (2017). MCSM-NA: Predicting the effects of mutations on protein-nucleic acids interactions. Nucleic Acids Res..

[40] Jubb H.C., Higueruelo A.P., Ochoa-Montaño B., Pitt W.R., Ascher D.B., Blundell T.L. (2017). Arpeggio: A web server for calculating and visualising interatomic interactions in protein structures. J. Mol. Biol..

[41] Pedregosa F., Varoquaux G., Gramfort A., Michel V., Thirion B., Grisel O., Blondel M., Prettenhofer P., Weiss R., Dubourg V. (2011). Scikit-learn: Machine learning in Python. J. Mach. Learn. Res..

[42] Doherty A.J., Jackson S.P. (2001). DNA repair: How Ku makes ends meet. Curr. Biol..

