# Supplementary material for: Uncovering the Molecular Drivers of NHEJ DNA Repair-Implicated Missense Variants and Their Functional Consequences
Source: Genes (Basel). 2023 Sep 29;14(10):1890. doi: 10.3390/genes14101890 (PMC10606680; doi:10.3390/genes14101890)
Supplement: Supplementary file 1 [file genes-14-01890-s001.zip › NHEJ_Supplementary Materials_v2.pdf]

**Supplementary Materials**

# Uncovering the Molecular Drivers of NHEJ DNA Repair-Implicated Missense Variants and Their Functional Consequences

**Raghad Al-Jarf [1,2,3], Malancha Karmakar [1,2,3], Yoochan Myung [1,2,3,4,] and David B. Ascher [1,2,3,4,*]**

[1] Structural Biology and Bioinformatics, Department of Biochemistry, University of Melbourne, Parkville, VIC 3052, Australia; mkarmakar@student.unimelb.edu.au (M.K.)
[2] Systems and Computational Biology, Bio21 Institute, University of Melbourne, Parkville, VIC 3052, Australia
[3] Computational Biology and Clinical Informatics, Baker Heart and Diabetes Institute, Melbourne, VIC 3004, Australia
[4] School of Chemistry and Molecular Biosciences, University of Queensland, St. Lucia, QLD 4072, Australia
* Correspondence: d.ascher@uq.edu.au (D.B.A.); Tel: +61-90354794 (D.B.A.)

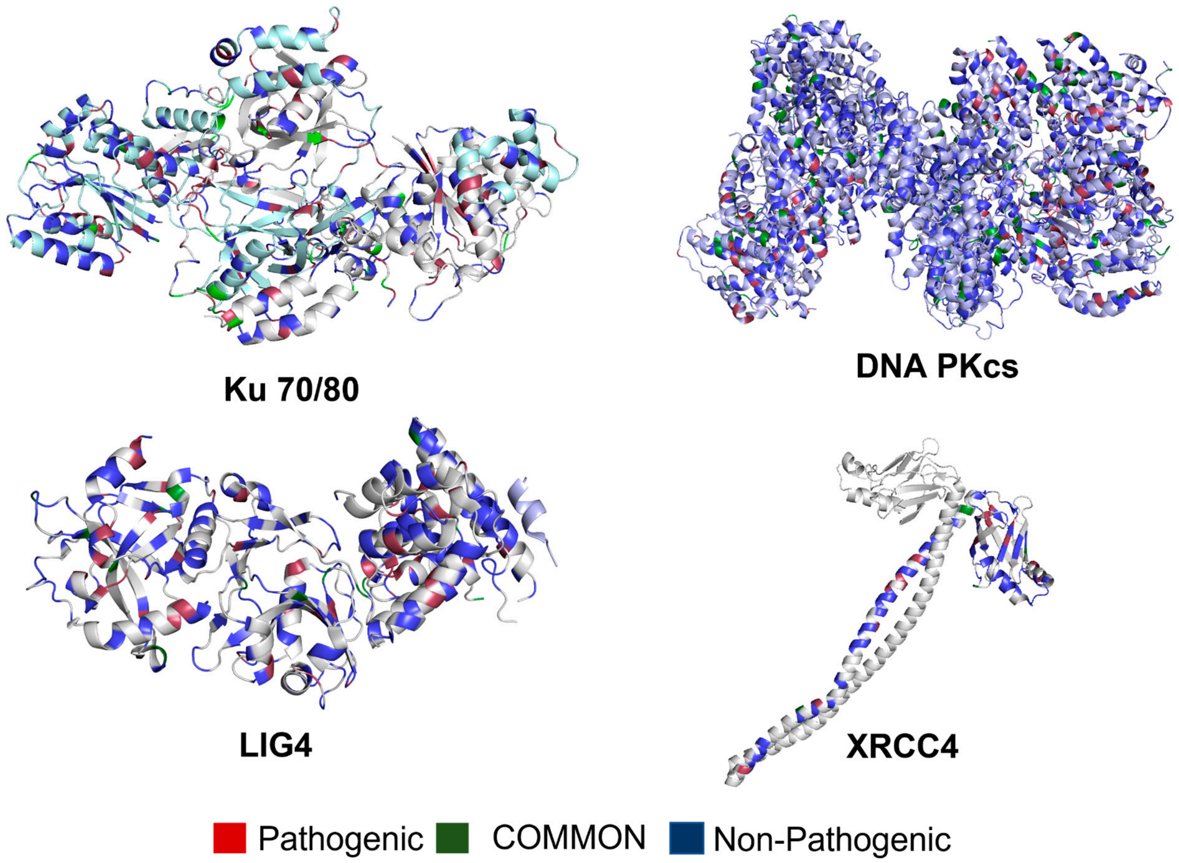


**Supplementary Figure 1. An overview of mutation distributions in NHEJ core components.** Different distributions of mutations across phenotypes: Pathogenic (red), and non-pathogenic mutations across the protein structures of NHEJ core components.

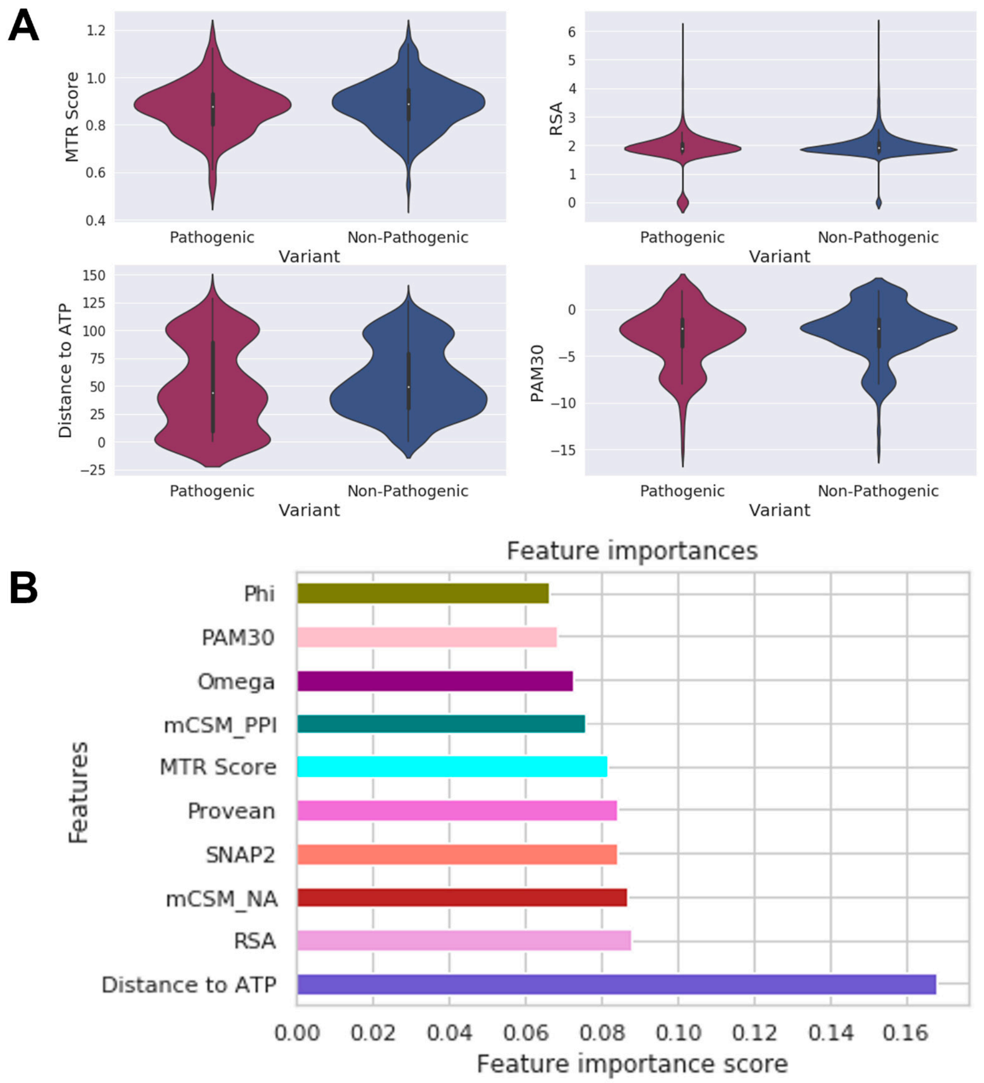


**Supplementary Figure 2. Main drivers of DNA-PKcs pathogenicity.** Based on statistically significant features identified by a Welch sample t-test (A), it was revealed that DNA-PKcs-mediated tumorigenesis is caused by changes in catalytic activities of DNA-PKcs, which are mediated by ATP, which was confirmed by supervised machine learning (B), where the distance to ATP, had the highest prediction capacity.

**Supplementary Table 1. NHEJ mutation curated database. (available as .xlsx file)**

**Supplementary Table 2. Mutation effects on DNA affinity in DNA-PKcs.** Mutations within DNA-PKcs regions 1503-1538, as generated in structure 5Y3R, were found to have three pathogenic and 10 non-pathogenic variants. Several of these mutations, including pathogenic mutations G1513E, L1510V and L1524P, increased the affinity for DNA. There was no apparent disparity between the two phenotypes, indicating that DNA affinity might not play an essential role in disease development.

| Mutation | Change in DNA Affinity (mCSM-NA) | Class |
|---|---|---|
| G1523R | 1.274 | Non-Pathogenic |
| G1513R | 1.18 | Non-Pathogenic |
| **G1513E** | **0.792** | **Pathogenic** |
| C1525R | 0.594 | Non-Pathogenic |
| S1506R | 0.634 | Non-Pathogenic |
| E1526K | 0.38 | Non-Pathogenic |
| **L1510V** | **0.042** | **Pathogenic** |
| A1518G | 0.068 | Non-Pathogenic |
| S1506T | 0.036 | Non-Pathogenic |
| L1505V | 0.1 | Non-Pathogenic |
| **L1524P** | **0.16** | **Pathogenic** |
| Q1509H | 0.088 | Non-Pathogenic |
| C1525G | 0.74 | Non-Pathogenic |
